# Passive immunization with polyclonal anti-SHIV IgG: partial protection or increased acquisition of heterologous tier 2 SHIV – depending on IgG dose

**DOI:** 10.1186/1742-4690-9-S2-P41

**Published:** 2012-09-13

**Authors:** AM Sholukh, NB Siddappa, V Shanmuganathan, SK Lakhashe, RA Rasmussen, JD Watkins, HK Vyas, MM Mukhtar, G Hemashettar, S Thorat, JK Yoon, F Villinger, FJ Novembre, G Landucci, DN Forthal, S Ratcliffe, M Robert-Guroff, V Polonis, DC Montefiori, HC Ertl, RM Ruprecht

**Affiliations:** 1 Dana-Farber Cancer Institute, Boston, MA, USA; 2 Yerkes National Primate Research Center and Emory University, Atlanta, GA, USA; 3 Division of Infectious Disease, University of California Irvine, Irvine, CA, USA; 4 University of Pennsylvania, Philadelphia, PA, USA; 5 Vaccine Branch, NCI, NlH, Bethesda, MD, USA; 6 The Military HIV Res. Program, Walter Reed Army Inst. of Research, Silver Spring, MD, USA; 7 Department of Surgery, Duke University School of Medicine, Durham, NC, USA; 8 The Wistar Institute, Philadelphia, PA, USA; 9 Dana-Farber Cancer Institute/Harvard Medical School, Boston, MA, USA

## Background

While passively administered broadly neutralizing monoclonal antibodies (bnmAbs) prevented SHIV acquisition, polyclonal Abs with high neutralizing titers provided only moderate protection in primates.

## Methods

We tested whether passive immunization with polyclonal IgG raised in rhesus monkeys (RMs) with chronic clade C SHIV infection, termed SHIVIG, could protect RMs against multiple low-dose intrarectal challenges with the R5 tier-2 SHIV-2873Nip carrying an HIV clade C envelope heterologous to the viruses/envelopes against which the IgG responses had been elicited. We compared in vitro SHIVIG characteristics with in vivo protection.

## Results

In vitro, SHIVIG demonstrated binding to SIV Gag, HIV Tat and Env of different clades, contained b12 and 4E10-like Abs and neutralized tier-1 and 2 viruses, including SHIV-2873Nip. NK-cell depletion decreased neutralizing activity in PBMC assays 20-fold. SHIVIG completely inhibited viral replication by ADCVI assay, but showed only 35% target-cell killing by ADCC assay.

Four groups of RMs were given SHIVIG at different doses: Group 1 (400 mg/kg), Group 2 (675 mg/kg), Group 3 (25 mg/kg) and Group 4 (none; virus-only control) followed by weekly low-dose challenges with SHIV-2873Nip. All controls and all SHIVIG-treated animals became systemically infected. RMs given 400 mg/kg of SHIVIG showed significantly lower peak viral RNA loads compared to controls. Surprisingly, single-genome analysis revealed a significant increase in the number of transmitted variants in Group 3 compared to controls (P=0.032), suggesting increased acquisition. Complement-mediated Ab-dependent enhancement of infection (C’-ADE) at low SHIVIG concentrations was observed in vitro.

## Conclusion

Lack of protection and possibly increased acquisition has been reported for a passive immunization study that tested the efficacy of HIV hyperimmune globulin in preventing infection in Ugandan infants born to HIV-positive women (Onyango-Makumbi, JAIDS 2011). Thus, our primate model data paralleled clinical phase III results and suggest that polyclonal anti-HIV-1 Abs play a dual role upon virus encounter.

